# Exploring what works well and less well in a community-based drop-in hub providing health and wellbeing services for people experiencing homelessness: a participatory action evaluation of service coordination

**DOI:** 10.1186/s12913-024-11897-x

**Published:** 2024-11-18

**Authors:** Emma A. Adams, Sheena E. Ramsay

**Affiliations:** Faculty of Medical Sciences, Population Health Sciences Institute, Newcastle University, Newcastle Upon Tyne, NE2 4AX UK

**Keywords:** Qualitative research, Homelessness, Health inequalities, Marginalised communities, Service coordination, Evaluation

## Abstract

**Background:**

People experiencing homelessness often face obstacles accessing health and social care support. Challenges are further exacerbated when support provision for multiple unmet needs are not integrated or coordinated. To overcome these challenges, there has been growing attention on integrating and co-locating health and wellbeing services for people experiencing homelessness. In an urban area of North East England, a long-standing Hub or ‘drop-in centre’ offers a range of health and wellbeing support by bringing together the different health and care system agencies in one space. However, little is known about the perspectives of providers on what works well and less well in how the different services are coordinated.

**Methods:**

Using a participatory action research approach, a qualitative service evaluation was undertaken between June and September 2023. Fourteen interviews were conducted with providers who work in a paid or voluntary capacity operating some of their service offerings or support in the Hub. Interview transcripts were analysed using inductive reflexive thematic analysis.

**Results:**

Three themes were evident from the evaluation: 1) location and space matter, 2) co-location and relationships make a difference, and 3) service consistency and flexibility are paramount.

**Conclusion:**

Co-locating support to cover the breadth of health and care needs has the potential to increase engagement and access for people experiencing homelessness, and to enhance trust with service users and between agencies. This model provides a unique example of co-location and integration of support, particularly with it being operated by a community housing organisation.

**Supplementary Information:**

The online version contains supplementary material available at 10.1186/s12913-024-11897-x.

## Background

Recent reports show that the needs of individuals experiencing homelessness are often multiple and complex, and housing alone is not the solution [1–4]. The intersection between housing instability, criminal justice, substance use, and physical and mental ill-health leads to many people who experience homelessness facing multiple and fragmented care and support pathways that do not always appropriately address all their needs. People experiencing homelessness are much more likely to have poor health outcomes, in terms of both physical and mental health, compared with the general population [5–7]. Often people experiencing homelessness face huge obstacles accessing health and social care support in their own communities [8–10]; obstacles that are harder to overcome when the support available is fragmented. Existing qualitative research with people experiencing homelessness has highlighted frustration with repeating stories across multiple agencies when services are disjointed or poorly coordinated [10].

A key cause of fragmentation is offering support at different locations with disconnected access processes [9, 11–13, 15]. The current evidence shows that coordinated and integrated access to care across the health, social care, housing, and criminal justice system is needed to support those experiencing homelessness [5]. One of the earliest solutions to promoting more integrated health, housing and social care needs for people experiencing homelessness was the Housing First initiative — integrating housing with specific health supports such as assertive community outreach [16–18] and harm reduction [19]. Housing First was first developed by Dr. Sam Tsemberis in New York to help people with mental ill-health living on the streets [16]. This initial model placed housing at the forefront, providing immediate access to an apartment. Notably, Housing First requires no behaviour modifications on the part of the person being helped, unlike many health care or traditional homelessness housing and support programs. Each Housing First housing offer was combined with access to a modified Assertive Community Treatment (ACT) team, which added a nurse for physical health needs and a housing support officer. Since its inception, Housing First has been implemented across the globe. Although much of the evidence on Housing First is focussed on housing outcomes, there is a substantial body of evidence focussed on health outcomes [17–21].

More recently researchers have been evaluating different coordinated, integrated and co-located models of access to health and social care for people experiencing homelessness [12, 13, 15, 22–25]. With the focus on health and wellbeing support, much of this research evidence reports on (and promotes) co-location of services often within primary care clinics [13], emergency departments [12, 15], or mental health or substance use services [23, 25]. There is limited research, however, looking to understand co-located or integrated models of health and social care situated in voluntary, community, or social enterprise (VCSE) organisations, or within housing organisations [24], such as community housing provider establishments. Given the role VCSE and housing organisations have in supporting people experiencing homelessness, it is important to consider and evaluate models based in these settings.

With national bodies such as the National Institute for Health and Care Excellence (NICE) [26], and National Institute for Health and Care Research (NIHR) [27] in England prioritising integrated and co-located health and wellbeing support for people experiencing homelessness, a better understanding of current models and approaches in England is imperative for identifying areas for shared learning to develop new and successful service coordination and delivery models. This paper explores the ways particular services come together in a community-based drop-in health and wellbeing Hub led by a community housing organisation in England as one contribution to the identified gap in the literature.

### Context and an overview of the Hub model

In the outskirts of Newcastle upon Tyne in North East England where there are high rates of homelessness, deprivation, and drug use, there is a Hub where health and wellbeing support is co-located for people experiencing homelessness. General and aggregated information about the Hub model have been provided below to situate the reader; however, specific details are not included to ensure anonymity for ethical requirements. This co-located Hub is led by a VCSE housing organisation, a community housing provider, and run out of a single building owned by this lead agency. The funding for this Hub is possible through a partnership between the local integrated health and care system (a partnership of organisations including local council, VCSE and community services that provide health and care across the region) and local council (which is responsible for public services in an area). However, the lead agency does not commission any of the providers who provide support within the Hub. Often funding for agencies offering support through the Hub comes from requirements linked to outreach or target key performance measures.

The Hub exists to ensure people who are experiencing or at risk of homelessness or not registered with primary health care have access to quality health and wider care support. People may not be registered with primary care because they recently moved into the area and have been unable to register, been refused by a clinic where there is not capacity for new enrolment or have disengaged from services due to negative experiences. Historically most of the visitors to the Hub are White British and currently or are at risk of experiencing some form of homelessness, ranging from sleeping on the streets (rough sleeping), staying with friends (couch surfing), sleeping in cars, or in temporary unstable accommodation or hostels. Over the last year, there has been an increased number of visitors who are seeking asylum or are currently in the country as a refugee. However, this has been temporary, is a small proportion of the overall visitors and has tended to fluctuate based on regional increases and decreases.

Providing a drop-in facility open Monday to Friday (and, recently, on the weekend), the Hub offers refreshments, food bank vouchers, bathing facilities, access to clothing, and access to a variety of health and wellbeing support delivered through external partnerships with local statutory and VCSE organisations. The lead agency (community housing provider), runs the psychosocial and wellbeing support and has staff members who are always in the facility, acting as the front reception and point of contact. External agencies offer outreach drop-in support on a weekly, biweekly, or monthly basis. Service offerings include, but are not limited to, primary health care, harm reduction, needle exchange, drug, alcohol, and mental health outreach, hepatitis C testing and treatment, and sexual health support. The most common reason visitors access the Hub is for support related to drug and alcohol use and mental health. The Hub undertakes internal evaluations to assess the satisfaction of visitors with having their needs met and the support available. Over the last five years the Hub has increased its partnerships with external agencies and there is a desire to continue growing its reach and impact. However, this will require a strong foundation to build and grow the working relationships across the various agencies involved.

### Aim

The purpose of this qualitative evaluation is to understand what is working well (and not so well) in the process of how services are coordinated in the co-located Hub. This evaluation focussed on the perspective of providers and agencies in order to understand their learning from implementation of this service coordination model. As the Hub model is still evolving, this evaluation sought to learn about how the implementation of service coordination could be improved, where learnings could be shared across the system, and opportunities for future development. The study did not include an evaluation of effectiveness outcomes (for example, health outcomes).

## Methods

Participatory action research places change or action at the forefront of the research process [28]. It is increasingly being used in public health and health research, which is unsurprising given the strength of the method in generating actionable solutions to improve health care [29]. A participatory action research lens was taken as it provided an approach for collaboratively undertaking the qualitative evaluation with the Hub lead agency. This lens also supported the development of knowledge that would be of direct relevance for the issues faced by the lead agency and focussed on generating knowledge that encouraged actionable learning for the Hub. The process undertaken as part of the evaluation outlined below leveraged the eight stages of participatory action research by Lloyd-Evans and colleagues at the University of Reading [30], and Table 1 presents examples of the different activities undertaken at each step and who was involved. Although the views of people accessing the service are important, the lead agency felt this was captured through internal service users’ evaluations and instead requested that this evaluation focussed on provider and agency perspectives on implementation of service coordination and potential areas for changes to improve the delivery of this new coordination model. While undertaking an embedded placement with the Hub, the lead author was approached by the lead agency for the Hub to undertake the evaluation. The overall aim and interview guide were developed collaboratively with Hub providers.

**Table 1:** Examples of activities and involvement undertaken at different stages of the participatory action research process

Stage^a^	Example of activities	People specifically engaged in activities from the Hub
1.Background	• Lead researcher undertook embedded placement in the Hub • Developed relationships with the agencies and providers working in the Hub • Reviewed internally commissioned and undertaken Hub evaluations	Lead agency frontline and management staff Agencies and providers operating within the Hub
2.Agreement	• Discussed existing gaps in knowledge for the Hub to continue development • Established an agreed scope for the evaluation based on the need identified by the Hub • Developed an agreement for who will be involved in the Hub at each stage of the evaluation • Applied for research funding to support with costs for transcriptions and presentation plans	Lead agency staff: senior management team member, manager of the Hub, and senior worker of the Hub
3.Choosing the questions	• Brainstormed and refined the aim and research questions that will be answered by the planned evaluation	Lead agency staff: senior management team member, manager of the Hub, and senior worker of the Hub
4.Research methods and data collection	• Identified purposive sampling criteria • Developed the preliminary topic guide and participant information sheets • Applied for research ethics approval • Lead researcher undertook interviews	Lead agency staff: senior management team member, manager of the Hub, and senior worker of the Hub
5.Data analysis	• Undertook analysis workshop to share preliminary analysis results and obtain feedback using anonymised quotes	Small workshop (n = 4): Lead agency staff and participant from the agencies and providers in the Hub
6.Key findings	• Identified key quotes for illustration • Provided final report headings and held discussion on content to include in reports • Provided feedback on initial versions of the draft report • Developed a visual communication of the early findings to increase the accessibility of findings	Lead agency staff: senior management team member, manager of the Hub, and senior worker of the Hub
7.Presentation	• Co-facilitated an in-person knowledge mobilisation workshop to get wider feedback on the findings from the evaluation and develop recommendation	Lead agency staff co-facilitated the workshop prep and presentation: CEO, senior management team member, manager of the Hub, and senior worker of the Hub Workshop (n = 50): Representation from commissioning in local government, agencies offering support in the Hub, researchers, public health practitioners, statutory health and social care providers
8.Action	• Worked with lead agency to finalise report and refine recommendations for the Hub to implement	Lead agency staff co-facilitated the workshop prep and presentation: CEO, senior management team member, manager of the Hub, and senior worker of the Hub

^a^These stages are based on the eight stages suggested by Lloyd-Evans and colleagues (2023) [30]

### Sampling and data collection

Between June and September 2023, interviews were conducted with service providers who work in a paid or voluntary capacity at the Hub. Verbal or written consent was obtained from participants before the interviews commenced. Purposive sampling was used to ensure that individuals from the different agencies based in the Hub were recruited. Staff employed or associated with the lead agency informed all external and internal providers about the study and shared the study information sheet and contact details of the lead researcher. All interviews were conducted by EAA, a female researcher (educated at MSc level) highly trained and experienced in qualitative data collection methods and analysis. EAA knew many of the participants through existing relationships with the agencies and the Hub, but confidentiality was reiterated along with the voluntary nature of the study. Interviews lasted on average half an hour and took place either online or in-person in a confidential space. The topic guide focussed on three main areas: 1) what is working well or not so well with how services are delivered, 2) recommendations for changes to the services, and 3) learnings for the wider health, social care, and homelessness support system. A copy of the semi-structured topic guide can be found in Supplemental Material 1.

People interviewed worked for organisations ranging from statutory services and speciality clinical support to third sector and voluntary organisations. Additionally, the lead agency staff members who were based in the Hub were interviewed. No incentives were provided to participants. To protect anonymity, specific characteristics of roles are not reported, and participant characteristics have been aggregated. Direct quotes have been lightly edited for flow. Human Research Ethics approval was provided by the Newcastle University Ethics Committee in May 2023 (Ref: 33,139/2023).

### Analysis

All interviews were audio-recorded and transcribed verbatim. An inductive reflexive thematic analysis was undertaken using NVivo 1.7.1 for Mac [31–33]. Analysis began by reviewing transcripts and notes for familiarisation, before generating codes for the data. Themes were constructed through combining, clustering and collapsing codes and then reviewed to ensure they captured the data. At this stage of the analysis, a small workshop (*n* = 4) was held to seek feedback from participants and people involved with operating the Hub to ensure the preliminary themes captured the experiences and perspectives of providers, while also having the opportunity to refine themes and subthemes. Themes were then refined prior to producing a report and recommendations for service coordination and system improvements. In line with the participatory action research lens, this report and recommendations were presented at a wider workshop with over 50 participants where we sought further feedback on the recommendations for both the Hub, and the wider health, social care, and homelessness support system. This step in the research led to production of a finalised report with clear recommendations for the Hub, which are currently being implemented.

## Results

Ten women and four men participated in interviews. Ages of participants ranged from 28 to 66 years (mean 51, standard deviation 11.6) and most people identified as White British. Specifics on demographic characteristics have been omitted to maintain anonymity. Three themes were evident from the data analysis: 1) location and space matter, 2) co-location and relationships make a difference, and 3) service consistency and flexibility are paramount.

### Location and space matter

Most providers agreed that locating the Hub in an area where there was high need both in terms of deprivation and current or risk of homelessness, was important for making it easily accessible. Providers explained:

*“I think it’s placed in a good place because you’ve got the hostel there. It’s a main street for homeless, for people with drug addictions and things like that. So it is in a good place, because a lot of them haven’t got money to get buses here, there and everywhere. So it’s in a really good spot to deliver the services you are delivering [speaking about themselves].”* (Provider 8)

*“It’s central. It gets a lot of footfall.”* (Provider 10).

The location was viewed positively as it increased the accessibility of the Hub for people experiencing homelessness.

When speaking specifically about the space of the Hub, providers said the environment felt relaxed and non-imposing. The fact that the lead agency was not a statutory health provider was viewed as a major benefit since often there was the perception that statutory services created environments that could feel intimidating for people experiencing homelessness. Such feelings were common among providers who worked for statutory health services and those who did not. As one provider explained:

*“It doesn’t feel as clinical which is a good thing. It just feels more welcoming. … It’s just not as intimidating because it’s not…people hear ‘NHS’ and they think, ‘Oh, we’re going to be stigmatised.’ So, yeah, less threatening. It’s less threatening…”* (Provider 1)

Despite these positives, privacy and confidentiality were highlighted as an area requiring further work in the current operating practices. The limited access to sound-proof, confidential and secure spaces meant that often providers felt that sensitive conversations could not take place. Providers shared:

*“Also, the person that’s using the telephone or the laptop or seeing [their worker] if they’re whispering, they’ve got confidentiality. If they’re not, then they haven’t because you can still hear what’s being said.”* (Provider 7)

*“Sometimes if that door is open and you are all in there, and they’re in this room here and they’re quite wary of what’s being said and things like that.”* (Provider 8)

Suggestions to resolve the issue of confidentiality included making better use of the separate rooms, shutting doors, and providing clear signage offering the opportunity to speak privately. As described by one provider:

*“…‘signage’, or saying, ‘If you need to speak privately, let a member of staff know.’ … But I think, yes, something as simple as making it known that if you need to have a private conversation that you can.”* (Provider 1)

Using the separate rooms was not without its risks, and a few providers had concerns around safety of staff if the available separate rooms were to be used more frequently. Changes to address these concerns could include switching the layout to ensure staff were closer to exits, installation of easily accessible safety alarms and clear procedures when working one-to-one in these spaces.

### Co-location and relationships make a difference

One of the biggest strengths of the Hub was that it brought services together from a range of health and social care agencies in one place. One provider explained: *“… whatever it is they need, it’s in one place.”* (Provider 9).

As a Hub access barriers faced by people experiencing homelessness were reduced, such as travelling across the city for appointments at different locations.

*“People don’t have to make multiple appointments in different places. I think, having everything in one place, where people are familiar with things, like, you don’t mind going to somewhere where you’re familiar with what the place is like, the staff that are there, you know, who you think’s going to be there. I think that would make it a lot more comforting for people, they’re a lot more likely to go, when they know what to expect almost, when they get there.” (Provider 12)*

Providers observed that they felt the central location worked well for visitors as visitors reported feeling more familiar and at ease with the Hub and support situated there. The visitors they supported were coming in more frequently, particularly as they could access the Hub for wellbeing support such as having a cup of coffee, something to eat, or a friendly conversation. The range of available support meant that there was some anonymity around which specific support visitors were accessing when they entered the building, with this seen as a positive compared with single-service focussed centres. A provider paraphrasing a visitor, for example, noted:

*“… it doesn't feel like I have to go there to see a GP or I have to go there for this. It feels like it's a bit of it an umbrella with things underneath that are going to help you.” (Provider 5)*

The Hub was recognised by providers as a natural gathering point for people experiencing homelessness in the region; a place to access support for their many and diverse needs or where they could find signposting to other agencies. The Hub being a gathering point was particularly important as providers felt that often health needs were deprioritised for people experiencing homelessness. Thus, if health services were available in places that catered to more pressing psychological needs like food, showers, warmth, and social connection, there was the chance that visitors would also access basic health care through treatment-oriented clinical health support. Additionally, co-location created opportunist connections with visitors who might not have otherwise engaged with specific agencies.

*“So, often, the visitors we see, tend to neglect their health. It might not be their biggest priority, but if it’s very easy to access, and they’re there for something else, picking up some food, or something else, then obviously, it’s quite easy for them to come in.” (Provider 3)*

*“The benefit is having those people there walking in and just catching opportunistically, catching people who would never probably attend a service like ours. It’s out there, people are walking in and we’re there for them.” (Provider 2)*

The co-location also often led to informal connections and new relationships between agencies. These connections were viewed positively for providers and particularly helpful in terms of their ability to support visitors.

*“Because I feel like even if it’s not necessarily sharing the information, it’s that whole networking and keeping those contacts with other agencies who, you may only speak to them on the phone or via email, and it’s nice to have that face-to-face contact and to build those relationships.” (Provider 4)*

*“Well, actually, because of us coming here, we’ve been trying to get into a certain [specific name removed for anonymity] service for a long, long, long, long time, and they wouldn’t share any information with us, even though we were both – We were supporting a lot of their patients ... Through us coming here and one of the homeless outreach workers for this particular service comes here as well, we’ve built up a relationship. Now we’ve got a meeting and they want to share information with us.” (Provider 10)*

Unfortunately, the informality of the partnerships between agencies often led to areas for improvement. One of the things that did not work so well was an awareness of all the support available and the expertise of each agency, which meant that sometimes support was not as joined up as it could be and remained fragmented. One provider explains:

*“Well, I’m not sure entirely, what all of the services are that are there.” (Provider 12)*

Another one went on to say:

*“And I suppose I’m aware of services coming in and out, but not so joined up in some ways, I suppose, really.” (Provider 13)*

Some suggestions for increasing awareness of services included holding forums for the providers to connect and providing up-to-date information on the specific support and the staff coming into the Hub and when. One provider suggested the use of a pictorial guide: *“…a photograph board”* where people could recognise *“‘You’re here today for this.’”* (Provider 2). The provider felt the photograph board could be useful for both providers and visitors.

Another area for improvement was data sharing between agencies. Providers highlighted that the lack of formal data sharing meant there was often a disconnect between agencies. One person explained:

*“… It would be better to have better information sharing between the services. I mean, I’m not sure what the best mechanism for doing that is because I think, at the moment, we all keep our own separate records.” (Provider 3)*

This was a frustration not unique to the Hub, but seen as a challenge across the service landscape. Data sharing was viewed as a missed opportunity when there are so many agencies coming together in one location. When asked for solutions, providers recognised the need for a systems approach to providing support to visitors.

### Service consistency and flexibility are paramount

The strength of trust and rapport built between visitors and providers led to a range of benefits. Providers felt visitors appreciated seeing familiar faces, and noted the relationship between visitors and providers promoted trust in their suggestions around other support to access, including within the Hub. The ability to build sustained relationships with visitors was a major facilitator to support delivery and coordination of multiple services and agencies. The rapport building helped encourage visitors to feel confident in asking for support and also being receptive to suggestions around accessing services.

*“Because the first time you go in, it might just be to get a cuppa, but then, once you know people’s faces, so, a lot of it’s building a rapport.”* (Provider 1)

*“I think it’s because you have probably the same people. So, the people that use us, do get used to seeing a familiar face.”* (Provider 9)

Another strength of the Hub delivery model in supporting ongoing access was the drop-in and flexible nature of the support offerings, which encouraged people experiencing homelessness to return on a regular basis. Many people explained the “*open-door access*” (Provider 13) was a core element that worked well. As one provider explained:

*“If they’re there, it’s a walk-in, if they want to come in and they’re there, we’ll see them straightaway rather than having to book appointments. These people are quite chaotic and it’s good to be there for them.”* (Provider 2)

The walk-in approach recognised the situation and circumstances of the visitors in ways traditional services that operated more on an appointment basis do not. The drop-in nature also allowed visitors to come in for a coffee or wellbeing-oriented support, which providers felt supported building ongoing confidence and trust in the Hub. It should be noted, however, that there were concerns that the drop-in nature and one person at a time at reception policy often meant there could be queues outside, which put visitors’ anonymity at risk. Safety concerns were also noted with the potential for arguments and disagreements to escalate when waiting outside.

## Discussion

Our evaluation found evidence to support implementing co-located community-based health and wellbeing support for people experiencing homelessness in VCSE or housing settings. Particular strengths for successfully bringing together services included operating from an accessible location, having a wide range of co-located health and wellbeing support, consistency in core staffing, and flexible service access. These strengths also led to perceived benefits in terms of better engagement and trust-building for people accessing the Hub as well.

This evaluation adds to the growing evidence base in England and more globally around integrated health and wellbeing support for people experiencing homelessness. Much of the evidence from North America has focussed on veteran homeless programmes [15, 22, 23], but such programmes tend to fall under a single commissioning and funding source rather than co-located programmes that need buy-in from different agencies. The lack of integration across the health and wellbeing agencies has led to challenges with data sharing and availability [12, 26]. Challenges linked with sharing data across different agencies, particularly in co-located models, is something echoed by our findings.

Several coordinated models focus on integrating specific health services only, such as emergency healthcare departments with primary care [22], mental health with primary care [23], and mental health with substance use [25]. Research evaluating models that integrate a variety of health support within community settings is more limited [24, 34]. Although both these studies offered drop-in models of support, they were only offered on specific days of the week. By contrast, the model we have evaluated operates Monday to Friday and has been trialling out weekend availability. In practice, there are likely several examples in England of co-located health and wellbeing models where the host is a charity or voluntary organisation and they partner with statutory health providers [24, 35, 36], however, there is limited empirical research on such models and learnings from implementing these approaches. Future research is needed comparing different models of co-located health and wellbeing support, which evaluate the longer-term benefits and cost-effectiveness of hosting co-located hubs in different settings.

### Strengths and limitations

This study is one of the only studies focussing on the coordination of support within a Hub. This study was not an evaluation of effectiveness of specific outcomes (for example, health outcomes). Additionally, with the growing recognition and appetite in England and globally around integrated and co-located care for people experiencing homelessness, this study provides much needed evidence for co-located services, where the host organisation is in the VCSE sector. A final strength to this project was its action-oriented and collaborative approach, which led to early identification of actionable insights for service changes and improvements, leading to early impact from the findings at a service and cross-system level.

At the request of the lead agency, this evaluation focussed on the perspective of only providers because of the need to learn from the implementation of the new ways of working within the Hub. This meant that a limitation of the study is that it did not include perspectives of service users (or visitors in the case of the Hub). Future research should include the direct perspective of service users on their experience of receiving service coordination models. Nonetheless, the findings from this evaluation still provides an important contribution to the evidence on coordination of support in co-located Hub models.

This study is limited by its focus on one specific co-located Hub in one region. This means not all of the findings will be relevant for other co-located models within England, the UK, or more globally, particularly where the host organisation may operate differently to a VCSE organisation. Having said this, the focus on agencies working together, rather than the specifics of service provision enables transferability of learning to other contexts. Furthermore, the regional workshop specifically included providers and policy makers who were not involved in the co-located Hub to ensure several of the recommendations for policy and practice were applicable to other settings.

Social desirability in the responses of participants is also a potential limitation of this evaluation that should be noted, as providers might have been less likely to share negative feedback. To mitigate this, confidentiality was emphasised and there were specific questions looking at where things didn’t work so well. Additionally, to unpack areas for improvement, the researcher used less negative language, such as ‘didn’t work so well’, after suggestions from developing and piloting the topic guide with the lead agency staff as this approach was perceived to encourage constructive conversations.

### Implications for practice

This evaluation emphasises the importance of accessibility in creating successful co-located health and wellbeing support for people experiencing homelessness. Support needs to be based in areas within communities with pre-existing high needs, which is echoed by other evidence [34, 37]. Health needs assessments for people experiencing homelessness and mapping of emergency and temporary accommodation offers can help identify high need regions. When designing and selecting buildings to host co-located hubs, creating non-stigmatising and fit for purpose spaces needs to be prioritised as this will ensure confidentiality and privacy for people accessing the service [10, 15]. Working with people who have lived experience of homelessness can help identify ways in which spaces can be better configured. Particular attention should be made to considering how spaces can take into consideration trauma-informed principles [38]. Future research should look to evaluate how well trauma-informed principles are embedded into the design, coordination and delivery of co-located health hubs for people experiencing homelessness. Additionally, the connotation associated with specific agencies for people experiencing homelessness cannot be ignored when deciding which agency should be the lead host for co-located hubs. A recent systematic review has emphasised the stigma and shame many people experiencing homelessness face when accessing services, which negatively impacts future access to support (38).

With the breadth of models that exist for co-locating health support for people experiencing homelessness, considerations need to be made for identifying the right agencies to bring together. Our evaluation demonstrates the importance of having a mix between traditional treatment-oriented health support and support focussed on more psychosocial needs such as hygiene, food and a warm space. These findings are echoed by another English evaluation, which found offering food brought people together [24]. Although it might not always be feasible for all agencies available in a co-located hub, where possible drop-in access models should be prioritised [34, 37]. Informal connections between agencies needs to be prioritised as an avenue for developing cross-service working relationships. This can be done in a variety of ways, from joint training events and ‘lunch and learns’ to bulletin boards introducing services and staff. Where it is possible, agencies need to prioritise consistency of staff, particularly for those who are at the initial point of access, to foster relationship building. Positive relationships between individual agencies within a hub as well as between those accessing the support and an individual service provider, improves continuity of care for people experiencing homelessness [9, 24].

This evaluation was undertaken with a view of identifying actionable change for the Hub and wider learnings for providing wrap-around care for people experiencing homelessness that could be implemented by others within this sector. Table 2 presents general recommendations for others looking to implement co-located health and wellbeing support for people experiencing homelessness in their local communities.

**Table 2:** Recommendations for implementing co-located health and wellbeing support for people experiencing homelessness

Target area for recommendations	Specific recommendations
Cross sector working	• Clear and transparent information that comprehensively covers different services available and when they operate • Create a partnership agreement, which provides common standards of practice for services operating within the co-located hub to encourage consistency across different services • Develop joint strategies around homelessness and health that span across services to enable better collaboration, cross-sector working, and ensure consistency in applying approaches to care • Create robust signposting and referral systems to ensure people can have continuity of care and access more speciality services when needed • Identify strategies to encourage better linkage of data across services, sectors, and systems
Optimising on space and the location	• Ensure access to separate, confidential spaces for different services to operate from, while keeping in mind the need for anonymity • Provide a single access point where services from across the health and social care system are available in an easily accessible location for people experiencing homelessness • Create co-located hubs in areas with the greatest need and along major transportation routes
Service offer	• Identify a minimum offer of health and wellbeing support that needs to be made available, ideally on a drop-in basis • Prioritise general wellbeing support, such as hydration and refreshments, to enable relationship building with people accessing support • Place lived experience at the centre of any new support offers to ensure that not only appropriate support is made available, but also that the support and the settings they take place in are accessible and non-judgmental
Continued education and learning	• Create a culture which encourages multidisciplinary team meetings to learn from best practices and identify better ways of working together (including data sharing on people’s needs) • Build on education and training to increase awareness and address the stigma, trauma, and disadvantage people experiencing homelessness face when accessing services • Host community learning events where leaders and people in communities can share about their work and successes and identify emerging challenges within the community • Leverage lived experience and peer support to continue shaping future service development

## Conclusion

Co-locating services to cover the breadth of health and care needs has the potential to increase engagement and access for people experiencing homelessness, whilst also providing an avenue to support relationship-building with service users and between agencies. This evaluation offered insight into an example of where things can work well when a range of services are delivered by different agencies, with some areas for further development. The evaluation provides the unique example of a community housing organisation leading this co-located health and wellbeing Hub, but further research could explore other models for co-locating and integrating a range of health, social care and wellbeing support for people experiencing homelessness. Agencies looking to design new models should carefully consider the location and physical space being used and identify ways to formalise partnerships to allow for clear routes for data sharing and collaboration between agencies with different data management and funding avenues.

## Supplementary Information

Supplementary Material 1.

## Data Availability

Due to the sensitivity of the topic and the number of participants, we are not able to share transcripts. Summaries are available from the corresponding author on request.

## Abbreviation

VCSE: Voluntary, community, social enterprise
