# Supplementary material for: Exploring what works well and less well in a community-based drop-in hub providing health and wellbeing services for people experiencing homelessness: a participatory action evaluation of service coordination
Source: BMC Health Serv Res. 2024 Nov 18;24:1423. doi: 10.1186/s12913-024-11897-x (PMC11572058; doi:10.1186/s12913-024-11897-x)
Supplement: Supplementary file 1 — Supplementary Material 1. [file 12913_2024_11897_MOESM1_ESM.docx]

**Project Title: Exploring what works well and less well in a community-based drop-in delivery model providing health and wellbeing services for people experiencing homelessness**

**Topic Guide**

**Introduction**

- Thank you for taking part in this study.
- The interview will last approximately 40 minutes, but please don’t feel rushed, take your time. You can have a comfort break, pause or terminate the interview at any time.
- Your participation is voluntary and you are able to stop the interview or decline to answer specific individual questions at any time should you wish.
- Go over information sheet and ask if they have any questions. Ask if they are happy to proceed.

**Confidentially and Consent**

- Remind about issues of confidentiality/ anonymity and data storage.
- Verbally confirm that they have read the information sheet and understand it.
- Complete consent forms, ensure everything is signed and dated.

Ask if they have any questions. Turn on audio recorder, if consent has been given.

**Questions**

***Introduction***

Q. Can you tell me a little bit about what you do at the Joseph Cowen Centre?

(Probes: role, organisations, how often at Joseph Cowen Centre, how long have you been offering services Joseph Cowen)

***What is working well and less well with the current offer?***

Q. Could you tell me a little bit about what you like about the delivery model at Joseph Cowen Centre?

Q. And is there anything you think works well about how health and care services are delivered in the Joseph Cowen Centre?

(Probes: location, people, accessibility, different services coming together)

Q. What maybe doesn’t work as well for how health and care services are delivered at the Joseph Cowen Centre?

(Probes: does anything help to overcome these issues? Is there anything you do not like about the centre?)

Q. Is there anything you would change about the delivery model at the Joseph Cowen Centre?

(Probes: Is there anything specific that you would suggest changing to ensure individuals who experience homeless are better able to access/use the service? Is there anything about how services work together/integrated?)

***Recommendations and moving forward.***

Tyne Housing is working with stakeholders to explore opportunities to expand the service delivery and reach of the Joseph Cowen Centre. We were hoping to get your thoughts on a few things.

Q. Thinking about your experience delivering services in the Joseph Cowen Centre, what makes this location/space different than some of the other places you might have previously or currently work(ed) in?

(Probes: space/location, service, people, commissioning, accessibility)

(Probes: are you aware of anywhere else that does something similar or better?)

Q. Thinking about how the service is delivered, is there any gaps in the current model or anything that could be changed?

(Probes: who would need to be involved? What would it look like? Where would it take place?)

Q. By contrast, if we were looking to replicate the service model used at Joseph Cowen Centre, what do you think would be the two most important elements we would need to get right?

(Probes: space/location, service, people)

Q. Perhaps more broadly, what would be the one or two things you think would be helpful for other services to learn based on how services are delivered at the Joseph Cowen Centre?

***Close***

Q. Is there anything that you think I have missed that you would like to add?

*Switch off recorder*
